# Association of environmental and socioeconomic indicators with serious mental illness diagnoses identified from general practitioner practice data in England: A spatial Bayesian modelling study

**DOI:** 10.1371/journal.pmed.1004043

**Published:** 2022-06-30

**Authors:** Joana Cruz, Guangquan Li, Maria Jose Aragon, Peter A. Coventry, Rowena Jacobs, Stephanie L. Prady, Piran C. L. White

**Affiliations:** 1 Department of Environment & Geography, University of York, Wentworth Way, York, United Kingdom; 2 Department of Mathematics, Physics and Electrical Engineering, Northumbria University, Newcastle-upon Tyne, United Kingdom; 3 Centre for Health Economics, University of York, York, United Kingdom; 4 Department of Health Sciences, University of York, York, United Kingdom; 5 York Environmental Sustainability Institute, University of York, York, United Kingdom; AUSTRALIA

## Abstract

**Background:**

The evidence is sparse regarding the associations between serious mental illnesses (SMIs) prevalence and environmental factors in adulthood as well as the geographic distribution and variability of these associations. In this study, we evaluated the association between availability and proximity of green and blue space with SMI prevalence in England as a whole and in its major conurbations (Greater London, Birmingham, Liverpool and Manchester, Leeds, and Newcastle).

**Methods and findings:**

We carried out a retrospective analysis of routinely collected adult population (≥18 years) data at General Practitioner Practice (GPP) level. We used data from the Quality and Outcomes Framework (QOF) on the prevalence of a diagnosis of SMI (schizophrenia, bipolar affective disorder and other psychoses, and other patients on lithium therapy) at the level of GPP over the financial year April 2014 to March 2018. The number of GPPs included ranged between 7,492 (April 2017 to March 2018) to 7,997 (April 2014 to March 2015) and the number of patients ranged from 56,413,719 (April 2014 to March 2015) to 58,270,354 (April 2017 to March 2018). Data at GPP level were converted to the geographic hierarchy unit Lower Layer Super Output Area (LSOA) level for analysis. LSOAs are a geographic unit for reporting small area statistics and have an average population of around 1,500 people. We employed a Bayesian spatial regression model to explore the association of SMI prevalence in England and its major conurbations (greater London, Birmingham, Liverpool and Manchester, Leeds, and Newcastle) with environmental characteristics (green and blue space, flood risk areas, and air and noise pollution) and socioeconomic characteristics (age, ethnicity, and index of multiple deprivation (IMD)). We incorporated spatial random effects in our modelling to account for variation at multiple scales.

Across England, the environmental characteristics associated with higher SMI prevalence at LSOA level were distance to public green space with a lake (prevalence ratio [95% credible interval]): 1.002 [1.001 to 1.003]), annual mean concentration of PM_2.5_ (1.014 [1.01 to 1.019]), and closeness to roads with noise levels above 75 dB (0.993 [0.992 to 0.995]). Higher SMI prevalence was also associated with a higher percentage of people above 24 years old (1.002 [1.002 to 1.003]), a higher percentage of ethnic minorities (1.002 [1.001 to 1.002]), and more deprived areas.

Mean SMI prevalence at LSOA level in major conurbations mirrored the national associations with a few exceptions. In Birmingham, higher average SMI prevalence at LSOA level was positively associated with proximity to an urban green space with a lake (0.992 [0.99 to 0.998]). In Liverpool and Manchester, lower SMI prevalence was positively associated with road traffic noise ≥75 dB (1.012 [1.003 to 1.022]). In Birmingham, Liverpool, and Manchester, there was a positive association of SMI prevalence with distance to flood zone 3 (land within flood zone 3 has ≥1% chance of flooding annually from rivers or ≥0.5% chance of flooding annually from the sea, when flood defences are ignored): Birmingham: 1.012 [1.000 to 1.023]; Liverpool and Manchester: 1.016 [1.006 to 1.026]. In contrast, in Leeds, there was a negative association between SMI prevalence and distance to flood zone 3 (0.959 [0.944 to 0.975]). A limitation of this study was because we used a cross-sectional approach, we are unable to make causal inferences about our findings or investigate the temporal relationship between outcome and risk factors. Another limitation was that individuals who are exclusively treated under specialist mental health care and not seen in primary care at all were not included in this analysis.

**Conclusions:**

Our study provides further evidence on the significance of socioeconomic associations in patterns of SMI but emphasises the additional importance of considering environmental characteristics alongside socioeconomic variables in understanding these patterns. In this study, we did not observe a significant association between green space and SMI prevalence, but we did identify an apparent association between green spaces with a lake and SMI prevalence. Deprivation, higher concentrations of air pollution, and higher proportion of ethnic minorities were associated with higher SMI prevalence, supporting a social-ecological approach to public health prevention. It also provides evidence of the significance of spatial analysis in revealing the importance of place and context in influencing area-based patterns of SMI.

## Abstract 2

In a cross-sectional study, Joana Cruz and colleagues study the relationship between environmental and socioeconomic factors and prevalence of serious mental illness in England between 2014 and 2018.

## Author summary

**Why was this study done?:**

- Serious mental illness (SMI), which includes schizophrenia, bipolar affective disorder, or psychosis, affects 335 million people worldwide.
- In England, the economic cost of SMI was estimated as £2.82 billion in 2019.
- The presence of environmental risk factors (e.g., air pollution concentration near the residential area) has been implicated in increased prevalence of SMI, but less is known about the relationship between residential green and blue space and prevalence of SMI.
- We wanted to know if the prevalence of SMI in England was associated with environmental conditions near the patient’s residential area, accounting for area-level socioeconomic characteristics.

**What did the researchers do and find?:**

- We developed an innovative mathematical model that included the average prevalence of patients diagnosed with schizophrenia, bipolar affective disorder and other psychoses, and other patients on lithium therapy at General Practitioner Practice (GPP) level for the period April 2014 to March 2018.
- The model included environmental variables near the patient’s residential area (i.e., area of woodland, public green space, distance to nearest public green space, distance to rivers or ponds within green spaces, distance to noise, distance to areas of high flood risk, and annual mean concentration of air pollution—particulate matter 2.5).
- The model also included socioeconomic variables that may be associated with SMI prevalence: percentage of ethnic minorities, age, and Index of Multiple Deprivation 2015.
- At national level, higher SMI prevalence was associated with greater distance from green spaces with lakes, higher levels of air pollution, and closeness to roads with high noise levels, although the significance and directionality of these associations varied between conurbations.

**What do these findings mean?:**

- Greater distance from green spaces with lakes is associated with higher prevalence of SMI, highlighting the complex interplay between green and blue spaces in urban neighbourhoods.
- Closer proximity to noise and air pollution is associated with higher prevalence of SMI, and these patterns are more pronounced among neighbourhoods with relatively higher measures of deprivation and in areas with more ethnic minorities.
- The patterning of the associations between green and blue space and prevalence of SMI varied between urban areas, suggesting that local variation is an important factor in understanding the impact of environment on mental health.

## Introduction

Serious mental illness (SMI), which includes schizophrenia, bipolar affective disorder, or psychosis, affects 335 million people worldwide [1] and is responsible for a significant health care burden. In England, the economic cost of SMI was estimated as £2.82 billion in 2019 [2]. People with SMI experience reduced life expectancy compared with the general population, e.g., for people diagnosed with schizophrenia, life expectancy is reduced by 13.6 (men) to 15.9 years (women) [3]. Recently, there has been a focus on the role of the environment on the risk of developing SMI. Research has shown that exposure to some air pollutants (e.g., NO_x_, NO_2_) during childhood is associated with increased prevalence of schizophrenia [4–6], while proximity to green spaces, blue spaces, and natural areas is associated with reduced rates of schizophrenia and other SMI [7–9]. But the evidence is sparse regarding the associations between environment and SMI in adulthood [10], as well as the potential links between these associations and the geographic distribution of SMI, the contextual factors that may affect these patterns [11].

Studies have linked road traffic noise with negative effects on the working memory and verbal domains in people with schizophrenia [12]. The World Health Organisation (WHO) recommends that average traffic noise should be below 53 dB, with adverse health effects if above this value, and noise becomes harmful when it exceeds 75 dB [13]. An association between air pollutants, such as particulate matter with less than 2.5 μm diameter (PM_2.5_), particulate matter with less than 10 μm diameter (PM_10_), ozone (O_3_), nitrogen oxides (NO_x_), sulphur dioxide (SO_2_), and health outcomes has been described often. For example, psychotic and mood disorders have been linked with long-term exposure to PM_2.5_ and NO_2_ [6,14,15], O_3_ [16], and seasonal peaks in NO_2_ [14]. The socioeconomic context of neighbourhoods also affects mental health [17]. Deprived neighbourhoods (high crime and education deprivation) have been associated with higher incidence of schizophrenia [18,19].

This paper evaluates the association between SMI prevalence and environmental characteristics in England. To guide our analysis, we adapted the framework developed by Zhang and colleagues [20] that combines the availability of green spaces, socioeconomic characteristics, with the context of neighbourhood, district boundaries, and urbanity, and combined the framework developed by Dzhambov and colleagues [21] (availability of blue spaces) with exposure to environmental stressors classified into man-made (air and noise pollution) and natural stressors (flood risk) according to their origin.

Firstly, we applied a fine-resolution spatial analysis at 2 levels—England as a whole, and its major conurbations—to allow for the identification of geographic patterns and any variability in the associations in different locations. We selected major conurbations because previous research suggests that prevalence of SMI in these areas is likely to be high [22], and they are more likely to experience poor environmental characteristics such as high noise, poor air quality, and limited availability of green and blue spaces [23].

Second, we evaluated, at Lower Layer Super Output Area (LSOA) level, the association between SMI prevalence and green and blue space, man-made stressors (noise and air pollution), natural stressors (flood risk), alongside socioeconomic factors (age, ethnicity, and deprivation), and compared the associations identified across England as a whole with those identified in each of the 5 major conurbations identified by Office National of Statistics [21,22]: Greater London, Birmingham, Liverpool and Manchester, Leeds, and Newcastle. We hypothesised that SMI prevalence would be lower in LSOAs with greater areas of green space and woodland, with shorter distance to green and blue space, greater distance from flooding zones, with lower pollution (air and noise) and in less deprived areas, with relatively older populations, and a higher percentage of ethnical minorities populations.

## Methods

We investigated the association between SMI mean prevalence, socioeconomic and environmental variables, by applying a Bayesian spatial regression model with random effects. This is a cross-sectional analysis of routinely collected and publicly available primary care data. The setting is in General Practitioner Practice (GPP) in England who submitted data between 2014/2015 and 2017/2018 to the NHS Quality and Outcomes Framework (QOF). The participants are aged 18+ and registered in those GPPs.

### Spatial level

Data were analysed at LSOA level. LSOAs are small areas designed to be of similar size, with an average of approximately 1,500 residents or 650 households. They were produced by the Office for National Statistics for the reporting of small area statistics, like the Census. The 32,482 LSOA units across England have a mean population of 1,500 individuals and no less than 1,000 people. Distance to environmental characteristics (e.g., green space, green space with a lake) were calculated as the Euclidean distance from the LSOA-weighted population centroid that reflects the spatial population distribution from the 2011 United Kingdom Census.

### Response variable: Mean serious mental illness prevalence

The response variable was the mean prevalence of SMI as defined by the QOF indicator MH001—people with a diagnosis of SMI: schizophrenia, bipolar or other affective disorders, and other patients on lithium therapy [24–28]. The QOF is an incentivized voluntary process for all GPP in England and was introduced as part of the GPP contract in 2004, detailing practice achievement results. The QOF contains 4 domains: Clinical, Public Health, Public Health—Additional Services, and Quality Improvement. Each domain consists of a set of achievement measures, known as indicators, against which practices score points according to their level of achievement. GPP are incentivised as part of the QOF payments to maintain this register which makes the recording of the indicator likely to be an accurate point prevalence estimate. Individuals who are exclusively treated under specialist mental health care and not seen in primary care at all were not included in this analysis. The QOF includes on average 97% of the active GPP in England and the number of patients ranged between 56,413,719 (April 2014 to March 2015) [25] and of 58,270,354 patients for financial year April 2017 to March 2018 [28] (S2 and S3 Tables). We used QOF data on SMI prevalence for the period April 2014 to March 2018, reported at the GPP level [24–28]. For each GPP, there are also data on the LSOA of origin of its registered patients [29]. The average SMI prevalence in an LSOA is a weighted average of the prevalence in the GPP where the inhabitants of that LSOA are registered; the weights are the proportion of patients from that LSOA registered in each of the GPP [30]. The mean prevalence was then taken for each LSOA for the period between April 2014 to March 2018. We chose to analyse the mean prevalence instead of annual data to provide more power to the response variable. There were 53 LSOAs that did not have values for the SMI prevalence in 2017/2018. Their outcomes were treated as missing and were imputed based on the covariate values of these LSOAs and the estimated random effects of the middle super output area (MSOA), District, and Clinical Commissioning Group (CCG) (i.e., groups of GPP which come together in each area to commission the best services for their patients and population) within which each of these LSOAs resides (see Statistical Analysis for more detail and definitions of MSOA and CCG).

### Environmental characteristics

To assess the relationship between SMI prevalence and environment, we considered variables that have been associated with health: green and blue space, flood risk areas, and air and noise pollution. We derived the following variables in relation to green space: area of public green space per LSOA (ha) [31], distance to the nearest point of access of public green space (km) [31], and woodland area (ha) in each LSOA [32] (see S4 Table).

Green and blue spaces are often associated with one another, and in this study, we included green spaces with water features (lakes and rivers) by measuring distance from the LSOA population-weighted centroid to the nearest public green space with a lake [33] and distance to a public green space with a river [34] (S4 Table). To calculate flood risk areas, we used the zoning with the highest probability of occurrence designated by the UK Environment Agency as Flood Zone 3 (i.e., land within this zone has ≥1% or 0.5% chance of flooding annually, from rivers and the sea, respectively) [35] (S4 Table).

We measured noise pollution exposure as the distance from the LSOA population-weighted centroid to the nearest source of automobile noise ≥75 dB (S4 Table). We used the Department for Environment, Food and Rural Affairs (Defra) dataset [36], which provides the annual average road noise levels for the 16-hour period between 7 AM and 11 PM, for 2017, in the following noise classes: 55 to 59, 60 to 64, 65 to 69, 70 to 74, >75 dB [36]. These data are only available for roads within areas with a population of at least 100,000 people and along major traffic routes. Therefore, not all of England has a noise map. In order to use this variable, we made the assumption that the areas not covered by this assessment did not have automobile noise ≥75 dB. For air pollution, we used Defra’s 1 × 1 km gridded modelled annual mean PM_2.5_ data for 2014 [37] (S4 Table). Defra makes use of the Automatic Urban and Rural Network, with 138 sites operating in 2014 to monitor and model at national scale PM_2.5_ roadside concentration. The reason for choosing this pollutant over any other was due to the existent literature that supports an association between PM_2.5_ and development of psychoses [6,14,15].

### Social, demographic, and economic factors

Socioeconomic variables were all measured at the LSOA level. We included ethnicity in our model since studies report that minority ethnic groups have higher incidence risk of SMI [19,34,35]. Ethnicity and age were both sourced from 2011 UK Census [38]. Ethnic minorities were measured as a percentage of the population in the following groups as identified by the 2011 UK Census [38]: Asian (Asian or British Asian), black (black, African, Caribbean, or black British), mixed (mixed or multiple ethnic groups; other ethnic groups). Adults (> = 18 years old) were split into 4 age groups: 18 to 24, 25 to 44, 45 to 64, ≥65 years old (S4 Table). We measured the percentage of the population in each age group.

To assess the association of socioeconomic variables with SMI prevalence, we used the scores of 4 domains (crime, barriers to housing and services, employment deprivation, and income deprivation) and 2 subdomains (living environment—indoors; education, skills, and training—adult skills) from the Index of Multiple Deprivation (IMD) 2015 [39] (S4 Table). The smaller the score, the less deprived the LSOA is. Each set of scores was transformed into quintiles with the first quintile being the least deprived category.

### Geographical variables

For geographical variables, we used geographic regions and settlement categories. The region indicator for the 9 regions in England—London, the North East, North West, Yorkshire, East Midlands, West Midlands, South East, East of England, and the South West—was included as a categorical covariate, as opposed to as a set of region-level random effects, due to a small number of regions. As discussed in Statistical Analysis, our model captures spatial variability in data via random effects specified at finer spatial resolution levels. Thus, the fixed effect specification on region is sufficient to account for regional differences. We used the following settlement categories: rural town and fringe; rural town and fringe in a sparse setting; rural village and dispersed; rural village and dispersed in a sparse setting; urban city and town; urban city and town in a sparse setting; urban major conurbation; urban minor conurbation, as defined by the Office of National Statistics [40,41] (S4 Table).

### Statistical analysis

There was no prospective protocol and the analysis plan was as follows. To investigate the association between SMI mean prevalence, socioeconomic and environmental variables, a Bayesian spatial regression model with random effects was constructed on the log-transformed mean SMI prevalence. Our model captures complex spatial dependency structures at different spatial resolution levels using spatial random effects. The Bayesian implementation of our spatial model enables us to flexibly construct and fit realistic models to describe the variability in SMI prevalence, to assess robustness of our conclusions to various plausible model assumptions, to incorporate uncertainty associated with the data and with the model parameters. Log transformation was applied to achieve normality for the distribution of the outcome values. Let *y*_*i*_ denote the log mean SMI prevalence of LSOA *i* (*i* = 1, …, *N* with *N* = 32482 LSOAs). Eq 1, referred to as the full model hereafter, models this outcome value *y*_*i*_ as a function of the risk factors and a collection of random effect terms.


$$y_{i}=\beta_{0}+\sum_{k=1}^{K}\beta_{k}x_{ik}+\;v_{MSOA[i]}+\;m_{District[i]}+\;g_{CCG[i]}+e_{i}$$ (1)

In Eq 1, *β*_0_ is the intercept. The term *x*_*ik*_ is the value of the *k*^*th*^ risk factor in LSOA *i* so the regression coefficient, *β*_*k*_, is the log prevalence ratio (PR) [42], measuring the effect of that risk factor on the outcome of interest, SMI prevalence. Also included in Eq 1 are 3 spatial random effect terms, *v*_*MSOA*[*i*]_, *m*_*district*[*i*]_, and *g*_*CCG*[*i*]_, specified at the MSOA (there are 6,791 in the study region), Local Authority District (District; 326), and CCG (207) levels, respectively. Each MSOA is formed based on a group of contiguous LSOAs. Among all MSOAs in England and Wales, the mean population size is 7,200 with the minimum of 5,000. Districts are administered by either single tier (e.g., Unitary Authority, the metropolitan district, and the London borough) or 2-tier local authorities (e.g., county and the local authority district) in various parts of England. CCGs are groups of GPP that come together in each area to commission the best services for their patients and population. These 3 sets of random effects were included in the model to capture the residual variability at the 3 geographical levels that was not accounted for by the inclusion of the observable covariates. Such residual variability can arise due to unmeasured/unobservable risk factors. Finally, *e*_*i*_ is the independent error term in the regression model and $e_{i}\sim N(0,\sigma_{e}^{2})$ for all LSOAs.

To fully specify the model in the Bayesian framework, prior distributions were assigned to the model parameters, which are the regression coefficients, the spatial random effects, and the random effect and the error precisions. The prior specifications are given as follows. For each regression coefficient, a vague normal prior with mean 0 and a variance of 1,000 (i.e., N (0, 1,000)) was assigned. The use of N (0, 1,000), in particular the large variance chosen, reflects the assumption that little is known about the association between each covariate and SMI prevalence. Therefore, the information used to estimate the regression coefficients largely comes from the data. For each set of spatial random effects, the Besag–York–Mollié (BYM) spatial prior model [43] was used. The BYM model is formulated as a sum of 2 sets of random effects, a set of spatially structured random effects and a set of spatially unstructured random effects. The spatially structured random effects are modelled via the intrinsic conditional autoregressive (ICAR) model. The ICAR model assumes that the random effects from 2 nearby spatial units at the same spatial resolution level (e.g., 2 MSOAs) are more like each other compared to the situation where these 2 spatial units are far apart. To operationalise the above idea of similarity in space, at each spatial level, we defined spatial proximity via contiguity whereby 2 areas (e.g., MSOAs) are neighbours to each other if they share a common boundary and they are not neighbours otherwise. These spatially structured random effects capture the residual variability that displays a spatial pattern. For the spatially unstructured random effects in the BYM model, the exchangeable model was used. This exchangeable specification on the random effects assumes that the effects from the unobserved/unmeasured covariates on SMI prevalence vary from one area to another but such varying effects do not display a spatial pattern. We also considered different versions of the full model, each with a different specification of the random effect component. Results on model comparison are summarised in S5 Table. Finally, a Gamma distribution, *Gamma*(1, 0.00005), was used as a vague prior on the error precision, $1/\sigma_{e}^{2}$, and on each of the random effect precisions associated with the BYM specification.

It is worth emphasising the following 2 points on the spatial modelling. First, under the ICAR specification, while spatial contiguity defines a local neighbourhood structure, spatial smoothing under the ICAR model is not restricted to an area’s immediate neighbours but spans and propagates throughout the small areas at that spatial level [44]. Second, estimation of the spatial random effects depends not only on the spatial prior model used but also on the observed small area SMI prevalence. A strength of the Bayesian approach is that we utilise both sources of information, prior and data, to estimate model parameters.

To gauge the contribution of each model component, we also fitted 2 models: the covariates only model and the random effects only model, the expressions of which are given in Eqs 2 and 3, respectively (Table 1). All terms are specified in the same way as for the full model.

$$y_{i}=\beta_{0}+\sum_{k=1}^{K}\beta_{k}x_{ik}+e_{i}$$ (2)

$$y_{i}=\beta_{0}+\;v_{MSOA[i]}+\;m_{District[i]}+\;g_{CCG[i]}+e_{i}$$ (3)

**Table 1:** Model comparison via DIC and WAIC.

Model	Covariates	Random effects at multi-spatial scales	DIC	WAIC
Covariates only	Yes	No	−12,006	−12,005
Random effects only	No	Yes	−64,855	−64,598
Full	Yes	Yes	−68,829	−68,562

Model comparison was performed via deviance information criterion [45] (DIC) and Watanabe–Akaike information criterion (WAIC) [46]. Both criteria evaluate models based on goodness of fit (how well a model describes the observed data) and model complexity. A smaller DIC or WAIC value indicates a better model (Table 1).

The analysis was placed within the Bayesian framework. This not only offers the flexibility to incorporate random effects at multiple spatial scales but also allows us to consider different plausible assumptions on the dependence structure of the random effects. The latter is important in terms of assessing potential sensitivity of our findings regarding the risk factor effects to different modelling assumptions. Parameter estimation for all models was carried out through the integrated nested Laplace approximation (INLA) approach via the R package R-INLA [47]. INLA, a well-established technique to implement Bayesian spatial models [48], has shown to be computationally efficient to handle the large number of spatial units (32482 LSOAs in England) and the complexity of our spatial model. Briefly, INLA obtains posterior estimation of parameters via the nested Laplace approximation, the defining feature of the method to enable fast computation for fitting complex models to large spatial datasets [49]. The Kullback–Leibler divergence (D_KL_), a standard output from INLA, is a diagnostic to measure the accuracy of the INLA approximation [50]. For the full model that we shall report in the Results section, the D_KL_ diagnostic values for all regression coefficients and all random effects were small, indicating a reliable fitting from INLA (D_KL mean_ = 9.666e^−05^, D_KL min_ = 0.000, D_KL max_ = 1.123e^−03^). S1 Fig shows the posterior distributions of the random effect standard deviations. All distributions are unimodal and well behaved where the distribution is not being pushed towards 0, indicating good estimations of these parameters.

For a covariate effect, we report the posterior mean and the 95% credible interval (formed using the 2.5^*th*^ and the 97.5^*th*^ percentiles of the posterior distribution) of the PR, i.e., exp(*β*_*k*_) with *β*_*k*_ being the regression coefficient in Eq 1. The posterior mean gives a point estimate of the covariate effect and the 95% credible interval, hereafter referred to as 95% CI, provides an interval estimate within which the “true” effect lies. An interval estimate that does not contain 1 indicates a high level of certainty (over 95% chance) that an association between the covariate in question and SMI prevalence exists—the value 1 indicates no association.

### Sensitivity analysis

The full model was run with 10 LSOAs excluded since some LSOAs had outlying outcome values and, for some, the outcome values were not described well by the full model. The effect estimates, however, were very similar to those presented in the Results section, showing the robustness of our findings. The 10 LSOAs are: Kensington and Chelsea 020A, Tower Hamlets 015A, Westminster 019B, Westminster 020C, Sefton 021D, Sheffield 036A, North Devon 002D, East Staffordshire 006B, Sheffield 073E, and Nottingham 026G.

Reporting of this study was done in accordance with STrengthening the Reporting of Observational studies in Epidemiology (STROBE) guidelines [51] (S1 STROBE Checklist).

This study used secondary analysis of routinely collected and anonymised clinical and census data and was exempt from HRA and institutional ethical review since it is considered low risk.

## Results

During the study period (2014/2015 to 2017/2018), the national average prevalence of patients diagnosed with schizophrenia, bipolar affective disorder, psychoses, and other patients on lithium therapy was 0.90%, reaching a maximum of 550,918 registered patients in 2017/2018. All major conurbations presented an average prevalence higher than the national average, with the highest mean values registered in Greater London (average (standard deviation, SD): 1.04% (0.33)) and Manchester and Liverpool (1.05% (0.27)) (Table 2).

**Table 2:** LSOA-level mean SMIs prevalence (% average (SD)) for the studied period (April 2014–March 2018) in England and major conurbations.

	National	Major conurbations				
	Mean prevalence (SD) (%)					
Study period	England	Greater London	Birmingham	Liverpool and Manchester	Leeds	Newcastle
April 2014–March 2018	0.90 (0.27)	1.04 (0.33)	0.98 (0.26)	1.05 (0.27)	0.96 (0.21)	0.95 (0.18)

LSOA, Lower Layer Super Output Area; SD, standard deviation; SMI, serious mental illness.

The descriptive statistics for environmental and socioeconomic variables are available in S6 Table.

### SMI mean prevalence model

The full model with the covariates and the random effects across 3 spatial levels gave the lowest WAIC and DIC, thus the best model. The full model was also the best among other versions with different random effect specifications (S5 Table). The second-best model, Version 4 in S5 Table, yielded covariate effects similar to as those from the full model, indicating the robustness of our findings on covariate effects against alternative model specifications. These 2 models only differ in the specification of the CCG level random effects where the full model (Version 1 in S5 Table) has the BYM model for the CCG random effects, and Version 4 (S5 Table) has the exchangeable model on these CCG random effects.

### Environmental characteristics

**Fig 1:**
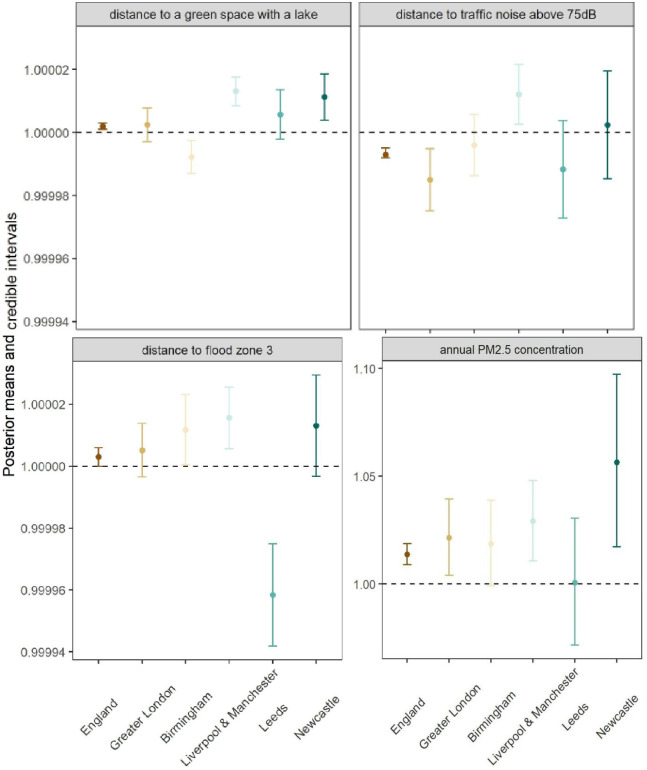
Posterior estimates (posterior mean and 95% credible interval represented by the dot and the vertical interval, respectively) of the PR of the environmental variables on mean SMI prevalence estimated from the full model. For all 4 covariates, the estimated covariate effect greater than 1 suggests that an LSOA with a larger covariate value (e.g., a higher annual PM_2.5_ concentration) was associated with a higher SMI prevalence. An estimate with its 95% CI excluding 1 indicates a high certainty that a covariate-outcome association exists. Note: The y axis plot for PM_2.5_ concentration is presented on a different scale to the other plots in the figure. LSOA, Lower Layer Super Output Area; PR, prevalence ratio; SMI, serious mental illness.

Four of the 8 environmental variables considered in the best model were found to be associated with SMI prevalence: distance to a public green space with a lake, distance to traffic noise ≥75 dB, distance to flood zone 3, and annual mean concentration of PM_2.5_ (Table 3 and Fig 1).

**Table 3:** Posterior estimates of the effects covariables and credible interval on mean SMI prevalence estimated from full model.

	England			Greater London			Birmingham			Liverpool and Manchester			Leeds			Newcastle		
	mean	0.0250	0.9750	mean	0.0250	0.9750	mean	0.0250	0.9750	mean	0.0250	0.9750	mean	0.0250	0.9750	mean	0.0250	0.9750
**Woodland area (ha)**	1.0000	1.0000	1.0000	0.9997	0.9993	1.0000	1.0000	0.9999	1.0001	1.0002	0.9999	1.0005	0.9998	0.9993	1.0002	1.0000	0.9998	1.0003
**Public green space (ha)**	1.0000	1.0000	1.0001	1.0000	0.9998	1.0002	1.0000	0.9998	1.0002	0.9999	0.9996	1.0001	1.0002	0.9997	1.0006	0.9996	0.9992	1.0000
**Distance to nearest public green space (km)**	1.0030	0.9980	1.0090	0.9782	0.9535	1.0035	0.9858	0.9577	1.0147	1.0124	0.9865	1.0388	0.9609	0.9174	1.0064	0.9786	0.9304	1.0293
**Distance to the nearest public green space with a lake (km)**	1.0020	1.0010	1.0030	1.0025	0.9971	1.0078	0.9922	0.9871	0.9975	1.0132	1.0085	1.0178	1.0057	0.9979	1.0136	1.0114	1.0039	1.0188
**Distance to the nearest public green space with a river (km)**	1.0000	0.9990	1.0000	0.9993	0.9976	1.0011	1.0011	0.9990	1.0031	1.0003	0.9985	1.0021	1.0006	0.9977	1.0035	0.9980	0.9950	1.0010
**Distance to noise ≥75 dB (km)**	0.9930	0.9920	0.9950	0.9851	0.9754	0.9948	0.9960	0.9864	1.0057	1.0122	1.0026	1.0218	0.9884	0.9732	1.0038	1.0024	0.9854	1.0197
**Distance to flood zone 3 (km)**	1.0030	1.0000	1.0060	1.0052	0.9965	1.0139	1.0119	1.0004	1.0234	1.0157	1.0057	1.0258	0.9593	0.9435	0.9753	1.0132	0.9967	1.0298
**Annual mean of particulate matter 2.5 (PM**_**2.5**_ **) (μg m-3)**	1.0138	1.0090	1.0186	1.0215	1.0039	1.0394	1.0187	0.9990	1.0388	1.0292	1.0107	1.0480	1.0006	0.9715	1.0305	1.0565	1.0172	1.0972
**Minority ethnic groups (Asian, black, mixed) (%)**	1.0015	1.0013	1.0017	1.0008	1.0004	1.0012	1.0014	1.0009	1.0020	1.0016	1.0011	1.0020	1.0017	1.0010	1.0023	1.0004	0.9992	1.0015
**18–24 years old (%)**	0.9967	0.9964	0.9971	0.9965	0.9954	0.9976	0.9953	0.9938	0.9967	0.9984	0.9973	0.9994	0.9903	0.9886	0.9920	0.9972	0.9955	0.9990
**25–44 years old (%)**	1.0024	1.0020	1.0028	1.0018	1.0008	1.0028	1.0030	1.0012	1.0047	1.0022	1.0010	1.0034	0.9993	0.9972	1.0014	1.0000	0.9977	1.0024
**45–64 years old (%)**	1.0027	1.0023	1.0032	1.0035	1.0019	1.0050	1.0030	1.0010	1.0049	1.0036	1.0022	1.0050	1.0025	0.9999	1.0051	1.0028	1.0005	1.0052
**≥65 years old (%)**	1.0009	1.0006	1.0012	0.9992	0.9981	1.0003	1.0005	0.9991	1.0018	1.0005	0.9995	1.0015	0.9982	0.9964	1.0000	0.9982	0.9964	1.0000
**Crime domain quintile [−0.69, -0.21]**	1.0069	1.0036	1.0102	1.0021	0.9802	1.0244	1.0120	0.9960	1.0283	1.0158	1.0026	1.0291	0.9771	0.9508	1.0041	1.0056	0.9886	1.0229
**Crime domain quintile [−0.21, 0.22]**	1.0138	1.0100	1.0176	1.0128	0.9907	1.0353	1.0108	0.9930	1.0290	1.0332	1.0189	1.0477	0.9686	0.9414	0.9967	1.0001	0.9804	1.0203
**Crime domain quintile [0.22, 0.69]**	1.0247	1.0204	1.0291	1.0291	1.0063	1.0524	1.0265	1.0069	1.0465	1.0413	1.0256	1.0573	0.9801	0.9507	1.0104	1.0233	0.9986	1.0486
**Crime domain quintile [0.69,3.28]**	1.0371	1.0320	1.0423	1.0403	1.0167	1.0645	1.0473	1.0254	1.0696	1.0491	1.0318	1.0667	0.9895	0.9574	1.0226	1.0430	1.0094	1.0777
**Income deprivation domain quintile [0.06, 0.09]**	1.0042	1.0005	1.0080	1.0220	1.0100	1.0342	1.0115	0.9912	1.0321	1.0029	0.9886	1.0175	1.0024	0.9801	1.0251	0.9921	0.9649	1.0201
**Income deprivation domain quintile [0.09, 0.14]**	1.0050	0.9999	1.0101	1.0395	1.0234	1.0559	1.0269	0.9991	1.0554	1.0112	0.9928	1.0299	0.9801	0.9484	1.0127	0.9953	0.9608	1.0310
**Income deprivation domain quintile [0.14, 0.23]**	1.0124	1.0057	1.0190	1.0524	1.0324	1.0728	1.0210	0.9866	1.0565	1.0219	0.9984	1.0459	1.0002	0.9582	1.0440	0.9865	0.9417	1.0333
**Income deprivation domain quintile [0.23, 0.64]**	1.0275	1.0188	1.0363	1.0704	1.0461	1.0953	1.0275	0.9873	1.0692	1.0340	1.0052	1.0636	1.0346	0.9822	1.0898	1.0140	0.9603	1.0707
**Barriers to housing and services domain quintile [12.3, 17.7]**	1.0070	1.0039	1.0102	0.9960	0.9752	1.0173	1.0100	0.9939	1.0264	1.0104	1.0020	1.0189	0.9969	0.9807	1.0134	0.9975	0.9808	1.0144
**Barriers to housing and services domain quintile [17.7, 23.1]**	1.0089	1.0055	1.0123	0.9983	0.9766	1.0206	1.0147	0.9978	1.0319	1.0098	0.9993	1.0203	0.9914	0.9734	1.0097	1.0052	0.9873	1.0235
**Barriers to housing and services domain quintile [23.1, 30.4]**	1.0144	1.0106	1.0181	1.0141	0.9912	1.0376	1.0203	1.0017	1.0391	1.0223	1.0089	1.0360	0.9901	0.9694	1.0111	1.0060	0.9858	1.0266
**Barriers to housing and services domain quintile [30.4, 72.6]**	1.0159	1.0113	1.0205	1.0057	0.9817	1.0303	1.0235	1.0009	1.0466	0.9942	0.9742	1.0146	0.9460	0.9150	0.9781	1.0195	0.9813	1.0591
**Employment deprivation domain quintile [0.06, 0.08]**	1.0139	1.0100	1.0178	1.0149	1.0026	1.0273	1.0220	1.0008	1.0437	1.0144	0.9996	1.0294	1.0275	1.0035	1.0520	1.0478	1.0184	1.0780
**Employment deprivation domain quintile [0.08, 0.12]**	1.0266	1.0216	1.0317	1.0300	1.0150	1.0453	1.0329	1.0058	1.0608	1.0233	1.0043	1.0427	1.0651	1.0313	1.1001	1.0655	1.0291	1.1033
**Employment deprivation domain quintile [0.12, 0.18]**	1.0358	1.0291	1.0426	1.0508	1.0322	1.0696	1.0555	1.0207	1.0915	1.0230	0.9984	1.0481	1.0718	1.0271	1.1185	1.0702	1.0237	1.1188
**Employment deprivation domain quintile [0.18, 0.58]**	1.0473	1.0386	1.0559	1.0781	1.0553	1.1013	1.0714	1.0319	1.1124	1.0302	1.0016	1.0597	1.0688	1.0176	1.1226	1.0811	1.0258	1.1395
**Indoors subdomain quintile [−0.74, −0.22]**	1.0012	0.9979	1.0045	0.9933	0.9808	1.0059	1.0026	0.9825	1.0231	1.0104	0.9982	1.0226	1.0274	0.9960	1.0597	0.9934	0.9768	1.0104
**Indoors subdomain quintile [−0.22, 0.23]**	0.9980	0.9943	1.0016	0.9927	0.9795	1.0059	1.0002	0.9795	1.0214	1.0029	0.9901	1.0158	1.0188	0.9865	1.0521	0.9905	0.9692	1.0123
**Indoors subdomain quintile [0.23, 0.74]**	1.0011	0.9970	1.0051	0.9981	0.9839	1.0124	1.0017	0.9800	1.0239	1.0046	0.9908	1.0186	1.0069	0.9754	1.0393	1.0104	0.9805	1.0413
**Indoors subdomain quintile [0.74, 3]**	1.0029	0.9981	1.0077	0.9967	0.9803	1.0133	1.0036	0.9803	1.0273	1.0075	0.9913	1.0239	1.0155	0.9812	1.0508	0.9408	0.8940	0.9899
**Adult skills subdomain quintile [0.21, 0.27]**	1.0067	1.0031	1.0104	0.9973	0.9870	1.0077	1.0134	0.9934	1.0338	1.0245	1.0101	1.0392	1.0172	0.9921	1.0429	1.0155	0.9890	1.0428
**Adult skills subdomain quintile [0.27,0.33]**	1.0085	1.0039	1.0131	0.9869	0.9741	0.9999	1.0293	1.0050	1.0541	1.0326	1.0145	1.0510	1.0229	0.9933	1.0533	1.0140	0.9832	1.0457
**Adult skills subdomain quintile [0.33, 0.4]**	1.0064	1.0008	1.0120	0.9884	0.9727	1.0043	1.0156	0.9880	1.0439	1.0316	1.0103	1.0534	1.0279	0.9922	1.0649	1.0069	0.9694	1.0459
**Adult skills subdomain quintile [0.4, 0.75]**	1.0136	1.0064	1.0208	0.9948	0.9748	1.0151	1.0343	1.0013	1.0683	1.0472	1.0215	1.0736	1.0300	0.9864	1.0756	1.0241	0.9793	1.0710

SMI, serious mental illness.

Some environmental variables showed differences in their patterns of association depending on the region and spatial scale. LSOAs with population-weighted centroids further away from a green space with a lake were found to be associated with higher SMI prevalence in England as a whole (PR [95% credible interval]): 1.002 [1.001 to 1.003] and in Manchester and Liverpool (1.013 [1.009 to 1.018]) and Newcastle (1.011 [1.004 to 1.019]). SMI prevalence in Birmingham presented the opposite association for green spaces with a lake (0.992 [0.987 to 0.997]) (Fig 1 and Table 3). LSOAs with increasing distance from roads with noise levels above 75 dB were associated with lower SMI prevalence in England (0.993 [0.992 to 0.995]) and Greater London (0.985 [0.975 to 0.995]), while the opposite association was found for Manchester and Liverpool (1.012 [1.003 to 1.022]) (Fig 1 and Table 3). LSOAs with population-weighted centroid further away from flood zones 3 were found to have lower SMI prevalence in Leeds (0.959 [0.943 to 0.975]) but LSOAs of the same feature in Birmingham (1.012 [1.00 to 1.023]), Manchester and Liverpool (1.016 [1.006 to 1.026]), and in England (1.003 [1.000 to 1.006]) were associated with higher SMI (Fig 1 and Table 3). Increasing annual mean concentration of PM_2.5_ was associated with higher SMI prevalence in all LSOAs at the national (1.014 [1.009 to 1.019]) and major conurbation scales (Greater London: 1.021 [1.004 to 1.039], Liverpool and Manchester: 1.029 [1.011 to 1.048], Newcastle: 1.056 [1.017 to 1.097]), except for Birmingham and Leeds areas, which showed no relationship (Fig 1 and Table 3).

The environmental covariates that showed no significant association with SMI prevalence were woodland area (England: 1.000 [1.000 to 1.000]; Greater London: 0.9997 [0.9993 to 1.000]; Birmingham: 1.0000 [0.9999 to 1.0001]; Liverpool and Manchester: 1.0002 [0.9999 to 1.0002]; Leeds: 0.9998 [0.9993 to 1.0002] and Newcastle: 1.0000 [0.9998 to 1.0003]); public green space area (England: 1.0000 [1.0000 to 1.0001]; Greater London: 1.0000 [0.9998 to 1.0002]; Birmingham: 1.0000 [0.9998 to 1.0002]; Liverpool and Manchester: 0.9999 [0.9996 to 1.0001]; Leeds: 1.0002 [0.9997 to 1.0006] and Newcastle: 0.9996 [0.9992 to 1.0000]); distance to the nearest public green space (England: 1.0030 [0.9980 to 1.0090]; Greater London: 0.9782 [0.9535 to 1.0035]; Birmingham: 0.9858 [0.9577 to 1.0147]; Liverpool and Manchester: 1.0124 [0.9865 to 1.0388]; Leeds: 0.9609 [0.9174 to 1.0064] and Newcastle: 0.9786 [0.9304 to 1.0293]); distance to the nearest public green space with a river (England: 1.0000 [0.9990 to 1.0000]; Greater London: 0.9993 [0.9976 to 1.0011]; Birmingham: 1.0011 [0.9990 to 1.0031]; Liverpool and Manchester: 1.0003 [0.9985 to 1.0021]; Leeds: 1.0006 [0.9997 to 1.0035] and Newcastle: 0.9980 [0.9950 to 1.0010]) (Table 3).

### Social, demographic, and economic factors

**Fig 2:**
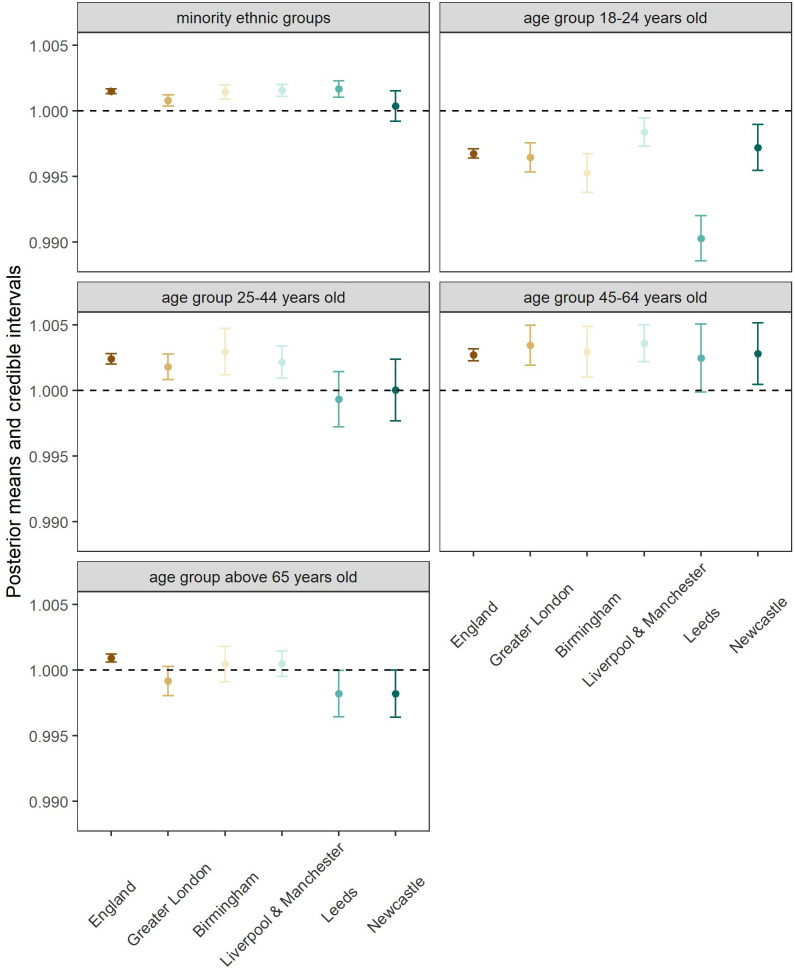
Posterior estimates (posterior mean and 95% credible interval represented by the dot and the vertical interval, respectively) of the PR of the socioeconomic variables on mean SMI prevalence estimated from the full model. For all 5 covariates, the estimated covariate effect greater than 1 suggests an LSOA with a larger covariate value (e.g., a higher percentage of minority ethnic groups) was associated with a higher SMI prevalence. An estimate with its 95% CI excluding 1 indicates a high certainty that a covariate-outcome association exists. LSOA, Lower Layer Super Output Area; PR, prevalence ratio; SMI, serious mental illness.

LSOA ethnic group and age composition were significantly associated with SMI prevalence in England and in at least one of the major conurbations (Fig 2 and Table 3). Except for Newcastle, LSOAs that had a higher percentage of minority ethnic groups showed higher prevalence of SMI (England: 1.001 [1.001 to 1.002]; Greater London: 1.001 [1.000 to 1.001]; Birmingham: 1.001 [1.001 to 1.002]; Liverpool and Manchester: 1.002 [1.001 to 1.002]; Leeds: 1.002 [1.001 to 1.002]) (Fig 2 and Table 3). In terms of age groups, the LSOAs with higher percentage of the youngest group in our analysis (18 to 24 years old) was associated with lower SMI prevalence, while LSOAs with higher percentage of older age groups were associated with higher prevalence rates nationally and in most conurbations (Fig 2 and Table 3). LSOAs with a high percentage of individuals aged between 25 to 44 years old presented high SMI prevalence at national level (1.002 [1.002 to 1.003]), and in Greater London (1.002 [1.001 to 1.003]), Birmingham (1.003 [1.001 to 1.005]), Manchester and Liverpool (1.002 [1.001 to 1.003]) (Fig 2 and Table 3). For the next age group, LSOAs with a high percentage of 45 to 64-year-old people, England (1.003 [1.002 to 1.003]) and all major conurbations (Greater London: 1.003 [1.002 to 1.005]; Birmingham: 1.003 [1.001 to 1.005]; Liverpool and Manchester: 1.004 [1.002 to 1.005]; Newcastle: 1.003 [1.000 to 1.005]), except Leeds, showed a positive association with SMI prevalence (Fig 2 and Table 3). A higher proportion of people above 65 years old in an LSOA was associated with a high SMI prevalence at national scale (England) (1.001 [1.001 to 1.001]), but not for individual conurbations (Fig 2 and Table 3).

**Fig 3:**
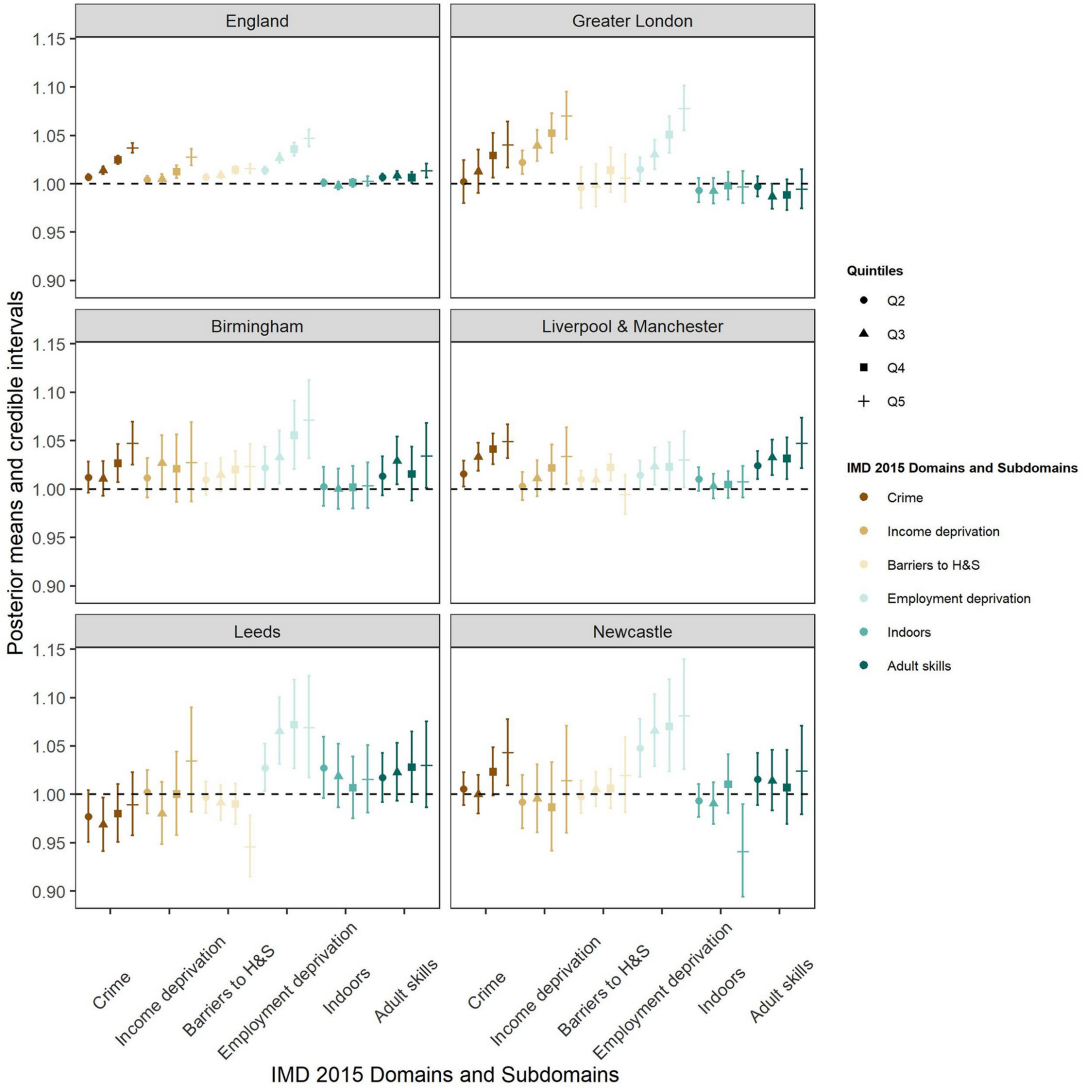
Posterior estimates of the effects across the quintiles of each IMD 2015 domains and subdomains on SMI prevalence at national level and major conurbations. For each index, the reference category was set at the least deprived group, and from left to right, from the less deprived to more deprived quintile. The solid dot represents the posterior mean of the estimated PR and the vertical bar shows the 95% CI. An estimate with its 95% CI covering the value 1 would indicate no difference in SMI prevalence between the corresponding category and the reference category. (Barriers to H&S: barriers to housing and services.) IMD, index of multiple deprivation; PR, prevalence ratio; SMI, serious mental illness.

For each of the 6 domains of the Index of Multiple Deprivation 2015 included in our model, the LSOA-level scores were categorised by quintile with the first category (the least deprived category) set as the reference. In terms of the domains and subdomains of the English Index of Deprivation, generally the most deprived areas were associated with higher SMI prevalence. This was the case in the crime, income deprivation, barriers to housing and services and employment deprivation domains, and adult skills subdomain (Fig 3 and Table 3). For the crime domain, England, Birmingham, Liverpool and Manchester, and Newcastle showed that the LSOAs with highest risk of crime had high SMI prevalence. Leeds was the only major conurbation where the SMI prevalence was not associated with the crime domain. The most deprived quintile of income deprivation was associated with high mean prevalence of SMI in England, Greater London, Liverpool, and Manchester when compared to least deprived quintiles on that domain (Fig 3 and Table 3). The most deprived quintiles of barriers to housing and services were associated with high levels of SMI prevalence in England (1.016 [1.011 to 1.021]), Birmingham (1.023 [1.001 to 1.047]), while Leeds showed the opposite relationship (0.946 [0.915 to 0.978]) (Fig 3 and Table 3). Employment deprivation was associated with the highest SMI prevalence when compared with the baseline (least deprived) at national and major conurbation scale, considering all the other domains and subdomains. LSOAs belonging to the most deprived quintile in this domain showed a positive association with SMI PR of 1.030 [1.002 to 1.060] (Manchester and Liverpool) to 1.081 [1.026 to 1.140] (Newcastle). For the indoors living environment subdomain, the levels of SMI prevalence did not differ across all the 5 categories at national level and for most of the major conurbations, with exception of Newcastle, where belonging to the most deprived quintile, presented an estimate PR of SMI in those LSOAs, of 0.941 [0.894 to 0.990] (Fig 3 and Table 3). SMI prevalence in England (1.014 [1.006 to 1.021]), Birmingham (1.034 [1.001 to 1.068]), and Liverpool and Manchester (1.047 [1.022 to 1.074]) was highest in the most deprived quintiles for adult skills scores (Fig 3 and Table 3).

We controlled for geographical variables, and at national level, the levels of SMI prevalence of all the urban categories were estimated to be considerably higher than that of the reference (the category of “Rural town and fringe in a sparse setting”), while the SMI prevalence showed no considerable difference among the rural categories. The PR was for the category of urban city and town in a sparse setting, with an increase of 1.146 [1.075 to 1.222] of the SMI PR compared with the reference (rural town and fringe in a sparse setting).

Table 4 summarises the variance partition coefficient, percentage of the total variability explained by each model component. The MSOA-level random effects were the most important component, accounting for over 51% of the total variability, in both the full models and the random effects only model. The covariates explained 15.6% of the total variability.

**Table 4:** Summary of the VPCs (posterior mean and 95% CI), percentage of the total variability explained by the 4 model components, the 3 sets of random effects and the covariates. Since the random effect only model does not include covariates, the VPC of the covariate component is 0%.

Component	Posterior mean of VPCs (95% CI)	
	The random effects only model (%)	The full model (%)
MSOA-level random effects	57.9 (46.9, 65.8)	51.0 (45.9, 56.4)
District-level random effects	22.6 (16.2, 31.1)	14.8 (11.9, 17.6)
CCG-level random effects	19.6 (11.7, 31.4)	18.5 (13.5, 25.6)
All covariates	N/A	15.6 (10.0, 25.4)

CCG, Clinical Commissioning Group; MSOA, middle super output area; VPC, variation partition coefficient.

**Fig 4:**
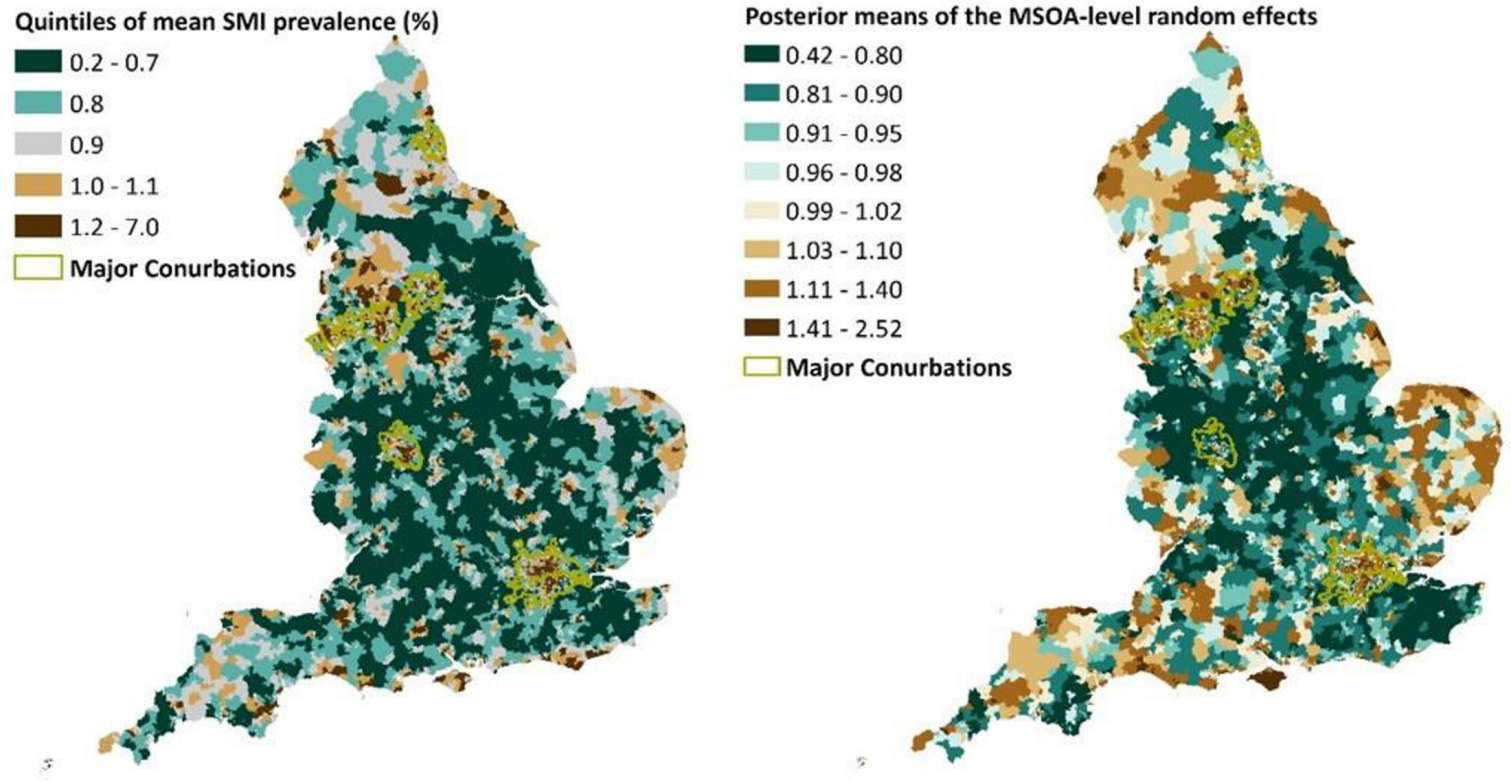
Mean SMI mean prevalence (quintiles) between financial year April 2014–March 2018 (left); posterior means of the MSOA-level random effects (right). (LSOA source: Source: Office for National Statistics licensed under the Open Government Licence v.3.0; Contains OS data Crown copyright and database right [2021].) LSOA, Lower Layer Super Output Area; MSOA, middle super output area; SMI, serious mental illness.

We visualised the posterior means of the MSOA-level random effects in Fig 4 (right). As a result, the posterior means of the MSOA-level random effects vary much more widely, from 0.42 to 2.52, compared to the district-level random effects (0.76 to 1.48) and CCG-level random effects (0.75 to 1.42). Fig 4 (right) also highlights clusters of areas with large posterior means: Cumbria, East Yorkshire and Humber coast, Suffolk and Norfolk, Greater London, parts of Devon and Cornwall, Isle of Wight, Dorset coast, and Lancashire. These areas also appear to have high SMI prevalence over the study period (Fig 4 (left)). This observation suggests that the levels of SMI prevalence in these areas remain high even after accounting for the socioeconomic and environmental factors in our model, pointing to the influence of unobserved or unmeasured factors.

## Discussion

In this study, we used Bayesian spatial models to explore spatial patterns in LSOA-level SMI prevalence and the association between SMI prevalence and socioeconomic and environmental factors in England and its major conurbations. For England as a whole, we found that higher SMI prevalence was associated with LSOAs further away from green spaces with lakes, closer to traffic noise and flood zones, with high levels of PM_2.5_, low percentage of people aged between 18 to 24 years old, high percentage of people aged between 25 and 64 years old, high percentage of ethnic minorities, more urban in character and with more deprived quintiles according to the domains and sub-domains of the Index of Multiple Deprivation 2015, namely crime, income deprivation, barriers to housing and services and employment deprivation domains, and adult skills domain. However, we observed variation in the associations between environmental characteristics and SMI prevalence in some of the major conurbations. In Birmingham, closeness to a green space with a lake was associated with high SMI prevalence. LSOAs in Liverpool and Manchester with greater proximity to traffic noise above 75 dB, and LSOAs in Birmingham, Liverpool, and Manchester with more areas with the greatest risk of experiencing flooding were associated with lower SMI prevalence. LSOAs in Leeds with the most deprived quintiles in barriers to housing and services, and LSOAs in Newcastle with the most deprived quintiles relating to the indoors living environment were associated with lower SMI prevalence.

### Comparison with previous literature

The results of the current study were consistent in some respects to the outcomes of other research but also provide new insights in others. In our study, we found no associations between SMI at area level with proximity to green spaces alone, but we did find associations with green spaces that also contained blue spaces. Very limited research has taken place considering the effect of blue spaces such as lakes, rivers, or canals on people with SMI. Recently, a few studies identified the protective effect of living nearby nature during childhood and lower risk of developing schizophrenia in adulthood [8,9,52–54]. Other observational studies have shown associations between exposure to green space, at individual [55] and neighbourhood level [56], or engagement in activities such as horticulture [57] with lower symptoms of schizophrenia. To the best of our knowledge, previous research is restricted to the absence of an effect for neighbourhood blue space on the length of hospital stay after a psychotic episode [58], and the protective effect during childhood against development of schizophrenia later in life [8,9,52–54]. Green spaces containing lakes, rivers, or canals may be more complex and biodiverse environments, which might provide more opportunities for psychological restoration and physical activity in general [59,60]. But an association between closeness to blue spaces and negative health outcomes, as we identified in Birmingham, has been identified before, in relation to increased risk of all-cause premature mortality [61] and higher anxiety/mood disorder hospitalizations [62]. The causes for these unexpected results are not clear but might be explained by possible exposure to pollutants and living in very deprived areas near water, which may reflect different historical developments of certain areas [63,64]. Some riverine areas in Birmingham are associated with high levels of pollutants (e.g., chromium, cadmium, lead, arsenic) [65,66] due to their association with historical industrial sites [67]. Some of these pollutants have been associated with the risk of developing schizophrenia, namely lead and chromium [68]. Other reasons might be related to study design and subjects (e.g., adults versus children). Most studies that look at the relationship of green and blue space on people with SMI are cross-sectional that makes it difficult to identify any causation between environmental exposure and SMI prevalence. Only recently, a longitudinal study following a cohort of children identified a dose–response association between the magnitude of green space during childhood and the risk of later development of schizophrenia [9,54]. Lastly, the numbers of metrics to measure blue and green space effect on health type are multiple, making it difficult to compare between different studies [69]. We used the Ordnance Survey’s data on public green spaces that classifies green spaces according to their function (public parks or gardens, play spaces, golf courses, sports areas or playing fields, churchyards or burial grounds, allotments, or community growing spaces) and excludes any other type of green space (private gardens, urban trees, etc.) [31]. Other authors have used normalized difference vegetation index (NDVI) [9,52,54,55], which provides information on vegetation density, and will therefore include public and private green spaces as well as urban trees, but provides no information regarding the function, structure, or quality of these spaces.

The opposite associations of noise pollution and proximity to flood zone 3 with SMI prevalence were also observed in Manchester and Liverpool (lower prevalence closer to flood zones and to high traffic noise) and Birmingham (lower prevalence closer to flood zones), when compared to national trends and described in literature. Noise has adverse effects on cognitive domains of individuals with schizophrenia [12], and being exposed to flooding is associated with mental health problems [70], and increased risk of hospital admission for schizophrenia [71]. The reasons for these major conurbations showing these unexpected patterns could be multiple and might be associated with urban and coastal gentrification [72]. In many towns and cities, areas near rivers and coastal regions, that are in flood risk zone 3 and formerly susceptible to flooding have been restored over the last few decades with improved flood protection measures, leading to a transition to wealthier residents.

Lastly, in terms of environmental characteristics, the association between PM_2.5_ with SMI prevalence we observed in this study is consistent with previous research. Air pollutants affect the brain in several ways [4], are responsible for the activation of inflammatory processes [73], and are linked with increased hospital admissions for mental illnesses [74–76], so it was expected that LSOAs with high levels of PM_2.5_ would report high SMI prevalence rates.

The socioeconomic associations with SMI prevalence that we identified largely confirm what has been described previously [77]. High SMI risk is associated with being unemployed, having low income, a low level of education [77–79], and living in areas with high crime rates [80]. SMI prevalence is higher in more deprived parts of England [81]. Peak age of onset of schizophrenia spectrum disorders is between 20 and 29 years old [82]. LSOAs with high percentage of age groups between 25 and 64 years old would therefore expect to have higher SMI prevalence. By comparison, LSOAs with high percentage of individuals above 65 years old are likely to report lower SMI prevalence, in part owing to reduced life expectancy up to 14.5 years [83] among people with SMI. Our investigation also identified an association between LSOAs with a relative higher percentage of ethnic minorities and higher SMI prevalence, as observed in other studies [84,85].

The rural–urban categories revealed a dual outcome. Firstly, urbanicity was associated with higher SMI prevalence when compared to rural areas. Previous epidemiological research has identified higher rates of serious and common mental health conditions in urban areas and stressed the need to address the increasing prevalence of mental illnesses associated with urbanisation and its expansion worldwide [86–88]. The same urban-rural effect was found in our study where LSOAs in the urban categories were estimated to have considerably higher SMI prevalence than those in the rural categories.

### Strengths and limitations

One of the strengths of our study was the social-ecological approach linking data across health, environment, and socioeconomic domains and modelling them at multiple spatial scales using a robust statistical approach [89]. This analysis provided important new information regarding associations of SMI prevalence with environmental characteristics at national scale, as well as highlighting important differences in these associations between major conurbations. The explanations could be multiple and need further investigation, but the findings of the present study suggest that each major conurbation has a set of particular characteristics and context that may be associated in a different way in their potential influence on SMI prevalence. Further research is needed to understand these differences, but this study provides an important first step in identifying areas to focus our attention and reiterates the importance of place-based solutions to prevent mental health problems. The other strength of the present study was the Bayesian spatial modelling approach that is flexible and powerful. This approach allowed us to construct realistic models to capture variability in SMI prevalence at different spatial resolutions, to examine different random effect structures to assess robustness of findings to different plausible model assumptions, and to incorporate various sources of uncertainty, from data (due to missing value) to unknown parameters, jointly.

One of the limitations of this study was the use of LSOAs as a unit of interest. LSOAs are useful to model health outcomes at a national scale but individual-level data provides further information regarding residence exposure and accessibility. Another limitation is related to the possible interactions that might exist between environment and socioeconomic factors and that were not explored at this stage. Deprived areas often lack green spaces [90–92] and their use might be limited due to concerns over safety [93], highlighting the importance of perceived accessibility alongside geographic accessibility, as well as broader social and economic inequalities. Our study used a cross-sectional approach, and as such, we were not able to draw casual inferences about our findings or investigate the temporal relationship between outcome and risk factors [94]. A few longitudinal studies have shown a negative association between the development of schizophrenia and growing up in rural areas or near green spaces [95,96]. There is scope for further longitudinal analyses of the relationship between environment and the onset and prevalence of SMI. Another limitation is the aggregation of the SMI prevalence. A spatial-temporal study would offer valuable insight regarding how the outcome changed over time. But there will be challenges to carry out such analysis, including developing appropriate space-time models and understanding and addressing data quality issues associated with yearly prevalence. Finally, the measurements of some of the covariates were from different years (e.g., woodland mapping and noise mapping in 2017; PM_2.5_ concentration in 2014; census data in 2011; IMD in 2015), while the health outcome was measured between 2014 and 2018. This may have an impact in our results due to changes in area or number throughout the years of these covariates that were not identified due to the lack of data availability for the same year.

### Implications for future research

An important finding was the lack of association between general measures of green space and SMI prevalence. This suggests the need to consider green space as a more complex variable than just distance to public green spaces or areas covered by them in each LSOA. An understanding of function, quality, area, assets, accessibility, and distribution around residence, neighbourhood and LSOA should be considered in future research [97]. Additionally, while SMI prevalence was mainly concentrated in urban areas, we observed that some rural areas (e.g., Cumbria, East Yorkshire and Humber coast, Suffolk and Norfolk, parts of Devon and Cornwall, Isle of Wight, Dorset coast and Lancashire) had high SMI prevalence, but the covariates included in our model were unable to capture these high levels of SMI prevalence. Mental health research has heavily focused on urban areas owing to the impact of rapid global expansion, higher levels of noise and air pollution, higher population density, and loss of social cohesion. Nevertheless, rural communities could also be at risk of suffering from mental illness, for example, suicide rates in these areas are higher than the average rate [98]. Rural populations are exposed to higher levels of ozone and pesticides, experience substantial inequalities in access to health and social care services, and community support [98]. Rural populations are also older than average and experience high rates of isolation, social exclusion, and high deprivation, leading to higher risk of mental health problems [98]. There is a need for more detailed modelling of the impact of environmental risk factors in rural areas on mental health.

## Conclusions

In conclusion, we used novel spatial analyses to map the relationship between a broad range of environmental and socieconomic factors and SMI prevalence. Our results provide further evidence on the significance of socioeconomic associations in patterns of SMI but emphasise the additional importance of considering environmental characteristics alongside socioeconomic variables in understanding these patterns. We showed that area-level associations between environmental factors and SMI prevalence were present for distance to green spaces that included lakes but were not significant for green spaces alone. However, this relationship varied between different urban conurbations, suggesting the importance of local variation in the configuration of green and blue spaces and their associations with mental health. Deprivation, higher concentrations of air pollution, and higher proportion of ethnic minorities were associated with higher SMI prevalence, supporting a social-ecological approach to prevention of mental ill health.

## Supporting information

S1 Table STROBE Statement for “The association between serious mental illness and environment and socioeconomic indicators in England: A spatial Bayesian modelling study”. (DOCX)

S2 Table Number of GPP, total number of patients registered, and number of patients diagnosed with SMI (schizophrenia, bipolar affective disorder and other psychoses, and other patients on lithium therapy) over the financial years of April 2014–March 2018 as reported by the QOF [25–28]. GPP, General Practitioner Practice; QOF, Quality and Outcomes Framework; SMI, serious mental illness.
(DOCX)

S3 Table Number of LSOAs, average number of GPP, and of patients over the financial years of April 2014–March 2018 for England and major conurbations as reported by the QOF [25–28]. GPP, General Practitioner Practice; LSOA, Lower Layer Super Output Area; QOF, Quality and Outcomes Framework.
(DOCX)

S4 Table Characterisation of the variables used to model SMI prevalence in England and major conurbations. SMI, serious mental illness.
(DOCX)

S5 Table Comparison of different versions of the full model. All versions here include covariates ^a^ but they have different specifications to the random effects. Version 1 is the full model presented in the main paper. BYM, Besag-York-Mollié spatial prior model; CCG, Clinical Commissioning Group; DIC, Deviance Information Criterion; District, Local Authority District; MSOA, middle layer super output area; WAIC, Watanabe–Akaike information criterion.
(DOCX)

S6 Table Characterisation of all the covariates included in the models (average (SD)) in England and major conurbations by LSOA. For explanation of the variables, see S4 Table. LSOA, Lower Layer Super Output Area; SD, standard deviation.
(DOCX)

S1 Fig Posterior distributions of the random effect SDs. CCG, Clinical Commissioning Group; District, Local Authority District; MSOA, middle layer super output area; SD, standard deviation.
(DOCX)

## Abbreviations

BYM: Besag–York–Mollié
CCG: Clinical Commissioning Group
DIC: deviance information criterion
GPP: General Practitioner Practice
ICAR: intrinsic conditional autoregressive
IMD: index of multiple deprivation
INLA: integrated nested Laplace approximation
LSOA: Lower Layer Super Output Area
MSOA: middle super output area
NDVI: normalized difference vegetation index
PR: prevalence ratio
QOF: Quality and Outcomes Framework
SD: standard deviation
SMI: serious mental illness
WAIC: Watanabe–Akaike information criterion
WHO: World Health Organisation
