# Supplementary material for: Association of environmental and socioeconomic indicators with serious mental illness diagnoses identified from general practitioner practice data in England: A spatial Bayesian modelling study
Source: PLoS Med. 2022 Jun 30;19(6):e1004043. doi: 10.1371/journal.pmed.1004043 (PMC9286217; doi:10.1371/journal.pmed.1004043)
Supplement: S2 Table — GPP, General Practitioner Practice; QOF, Quality and Outcomes Framework; SMI, serious mental illness. (DOCX) [file pmed.1004043.s002.docx]

Supplementary Material

S2 Table – Number of General Practitioner Practices, total number of patients registered and number of patients diagnosed with serious mental illness (SMI) (schizophrenia, bipolar affective disorder and other psychoses and other patients on lithium therapy) over the financial years of April 2014-March 2018 as reported by the Quality and Outcome Framework (QOF) [1-4].

| Financial Year | Number of General Practitioner Practices included in QOF | Number of patients registered | Number of patients diagnosed with SMI |
| --- | --- | --- | --- |
| April 2014 – March 2015 | 7,997 | 56,413,719 | 500,451 |
| April 2015 – March 2016 | 7,859 | 56,971,330 | 518,320 |
| April 2016 – March 2017 | 7,680 | 57,587,598 | 534,431 |
| April 2017 – March 2018 | 7,492 | 58,270,354 | 550,918 |
| TOTAL |  | 229,242,901 | 2,104,120 |

## References

1. Primary Care Domain, NHS Digital. Quality and Outcomes Framework - Prevalence, Achievements and Exceptions Report – Technical Annex 2017-18. In: Centre HaSCI, editor.: NHS Digital; 2018. p. 16.

2. Primary Care Domain, NHS Digital. Quality and Outcomes Framework – Prevalence, Achievements and Exceptions Report, England 2015-16. In: Centre HaSCI, editor.: NHS Digital; 2016. p. 43.

3. Primary Care Domain, NHS Digital. Quality and Outcomes Framework – Prevalence, Achievements and Exceptions Report – Technical Annex 2016-17. In: Centre HaSCI, editor.: NHS Digital; 2017. p. 15.

4. Primary Care Domain, Health Social Care Information Centre. Quality and Outcomes Framework – Prevalence, Achievements and Exceptions Report, England 2014-15. NHS Digital; 2015. p. 55.
