# Supplementary material for: Association of environmental and socioeconomic indicators with serious mental illness diagnoses identified from general practitioner practice data in England: A spatial Bayesian modelling study
Source: PLoS Med. 2022 Jun 30;19(6):e1004043. doi: 10.1371/journal.pmed.1004043 (PMC9286217; doi:10.1371/journal.pmed.1004043)
Supplement: S3 Table — GPP, General Practitioner Practice; LSOA, Lower Layer Super Output Area; QOF, Quality and Outcomes Framework. (DOCX) [file pmed.1004043.s003.docx]

Supplementary Material

Table S3 – Number of Lower Layer Super Output Areas (LSOAs), average number of General Practitioner Practices and of patients over the financial years of April 2014-March 2018 for England and major conurbations as reported by the Quality and Outcome Framework [1-4].

| National and major conurbations | Number of LSOAs | Number of General Practitioner Practices (range) | Number of patients registered  (range) |
| --- | --- | --- | --- |
| England | 32,843 | 7,492 - 7,997 | 56,413,710 – 58,270,354 |
| Greater London | 5,629 | 1,883 – 2,116 | 10,446,280 – 11,047,466 |
| Birmingham | 1,581 | 559 - 669 | 2,755,809 – 2,864,063 |
| Liverpool & Manchester | 2,515 | 884 - 998 | 4,218,753 – 4,351,102 |
| Leeds | 1,061 | 348 - 426 | 1,850,105 – 1,906,874 |
| Newcastle | 737 | 237 - 263 | 1,189,749 – 1,211,576 |

## References

1. Primary Care Domain, NHS Digital. Quality and Outcomes Framework - Prevalence, Achievements and Exceptions Report – Technical Annex 2017-18. In: Centre HaSCI, editor.: NHS Digital; 2018. p. 16.

2. Primary Care Domain, NHS Digital. Quality and Outcomes Framework – Prevalence, Achievements and Exceptions Report, England 2015-16. In: Centre HaSCI, editor.: NHS Digital; 2016. p. 43.

3. Primary Care Domain, NHS Digital. Quality and Outcomes Framework – Prevalence, Achievements and Exceptions Report – Technical Annex 2016-17. In: Centre HaSCI, editor.: NHS Digital; 2017. p. 15.

4. Primary Care Domain, Health Social Care Information Centre. Quality and Outcomes Framework – Prevalence, Achievements and Exceptions Report, England 2014-15. NHS Digital; 2015. p. 55.
