# Supplementary material for: Association of environmental and socioeconomic indicators with serious mental illness diagnoses identified from general practitioner practice data in England: A spatial Bayesian modelling study
Source: PLoS Med. 2022 Jun 30;19(6):e1004043. doi: 10.1371/journal.pmed.1004043 (PMC9286217; doi:10.1371/journal.pmed.1004043)
Supplement: S4 Table — SMI, serious mental illness. (DOCX) [file pmed.1004043.s004.docx]

Supplementary Material

S4 Table - Characterisation of the variables used to model serious mental illness prevalence in England and major conurbations. (LSOA, Lower Layer Super Output Areas; QOF, Quality and Outcome Framework)

| Variables | Definitions and characterisation |
| --- | --- |
| **Outcome** |  |
| Mean Serious Mental Illness (SMI) prevalence (%) | average prevalence of patients diagnosed with schizophrenia, bipolar affective disorder and other psychoses and other patients on lithium therapy (code MH001 from QOF 2014/15-2017/18) |
| **Environmental variables** |  |
| Woodland (ha) | Patches of woodland and forest that are over 0.5 ha, minimum of 20% canopy cover and minimum width of 20 metres (Source: National Forest Inventory Woodland (NFIW) GB 2017 [1] |
| Public green space (ha) | They include allotments, bowling green, cemetery, religious grounds, golf course, play space, playing field, tennis court, other sports facility and public park or garden (Source: OS Open Greenspace) [2] |
| Distance to nearest public green space (km) | Distance from the LSOA population weighted centroid to the nearest point of access of public green space (Source: OS Open Greenspace) [2] |
| Distance to the nearest public green space with a lake (km) | Distance from the LSOA population weighted centroid to the nearest point of access of public green space with a lake (Source: Water Framework Directive Lake Waterbodies Cycle 2 shapefile) [3] |
| Distance to the nearest public green space with a river (km) | Distance from the LSOA population weighted centroid to the nearest point of access of public green space with a river (Source: OS Open Rivers) [4] |
| Distance to noise ≥75dB (km) | Distance from the LSOA population weighted centroid to the nearest source of automobile noise ≥75 dB. We used DEFRA’s dataset, which provides the annual average road noise levels for the 16-hour period between 7 am and 11 pm, for 2017 ^39^. These data are only available for roads within areas with a population of at least 100,000 people and along major traffic routes (Source: DEFRA) [5] |
| Distance to flood zone 3 (km) | Distance from the LSOA population weighted centroid to flood zone 3, zoning with the highest probability of occurrence (i.e. land within this zone has ≥1% or 0.5% chance of flooding annually, from rivers and the sea, respectively) [6] |
| Annual mean of particulate matter 2.5 (PM_2.5_) (μg m^-3^) | The annual mean of PM_2.5_ was derived from Defra's Modelling of Ambient Air Quality for 2014 [5] by using the Inverse Distance Weighted interpolation method in ArcGis 10.3.1 |
| **Sociodemographic-economic indicators** |  |
| Minority ethnic groups (Asian, Black, mixed) (%) | All ethnic groups (Asian or British Asian; Black, African, Caribbean or Black British; Mixed or Multiple ethnic groups; other ethnic groups) as used in the 2011 Census of England and Wales [7] |
| Age groups | Age groups calculated from 2011 Census of England and Wales [7] |
| 18-24 years old (%) |  |
| 25-44 years old (%) |  |
| 45-64 years old (%) |  |
| ≥65 years old (%) |  |
| Index of Multiple Deprivation 2015 (scores) | As calculated by the Department for Communities and Local Government [8] |
| Crime domain | rate of violence, burglary, theft and criminal damage per 1,000 at risk population and/or properties |
| Income deprivation domain | the proportion of the population in an area experiencing deprivation due to low income |
| Barriers to housing and services domain | physical and financial accessibility of housing and local services |
| Employment deprivation | measures the proportion of the working-age population in an area involuntarily excluded from the labour market |
| Indoors subdomain | Measures the quality of housing |
| Adult skills subdomain | measures the lack of qualifications in the resident working-age adult population. |
| **Geographic** |  |
| Region |  |
| London |  |
| North East |  |
| North West |  |
| Yorkshire |  |
| West Midlands |  |
| East Midlands |  |
| South East |  |
| East of England |  |
| South West |  |
| Urban and rural areas | As defined by Office National of Statistics [9, 10] |
| Rural town and fringe in a sparse setting |  |
| Rural village and dispersed |  |
| Rural village and dispersed in a sparse setting |  |
| Urban city and town |  |
| Urban city and town in a sparse setting |  |
| Urban major conurbation |  |
| Urban minor conurbation |  |

## References

1. National Forest Inventory Woodland GB 2017. Forestry Commission. 2018 [cited 04/12/2019]. Available from: <https://data.gov.uk/dataset/f316113c-acdf-445b-8576-2bd87e81bf17/national-forest-inventory-woodland-gb-2017>.

2. OS Open Greenspace. Ordnance Survey Limited. 2018 [cited 20/05/2019]. Available from: <https://digimap.edina.ac.uk/webhelp/os/osdigimaphelp.htm#data_information/os_products/os_open_greenspace.htm>.

3. WFD Lake Waterbodies Cycle 2. Environment Agency. 2019 [cited 20/05/2019]. Available from: <https://data.gov.uk/dataset/da4a6c1f-7349-4d0e-9d21-8c90aa387b54/wfd-lake-waterbodies-cycle-2>.

4. OS Open Rivers. Ordnance Survey Limited. 2018 [cited 09/05/2019]. Available from: <https://digimap.edina.ac.uk/webhelp/os/osdigimaphelp.htm#data_information/os_products/os_open_rivers.htm>.

5. Defra. Strategic noise mapping (2017): Department for Environment Food & Rural Affairs; 2017 [cited 2019 02/04/2019]. Available from: <https://www.gov.uk/government/publications/strategic-noise-mapping-2019>.

6. Environment Agency. Flood Map for Planning (Rivers and Sea) - Flood Zone 3: Environment Agency; 2018 [cited 2019 13/05/2019]. Available from: <https://data.gov.uk/dataset/bed63fc1-dd26-4685-b143-2941088923b3/flood-map-for-planning-rivers-and-sea-flood-zone-3>.

7. 2011 Census aggregate data. UK Data Service. 2016 [cited 08/05/2019]. Available from: <http://dx.doi.org/10.5257/census/aggregate-2011-1>.

8. Smith T, Noble M, Noble S, Wright G, McLennan D, Plunkett E. The English indices of deprivation 2015. London, UK: Department for Communities and Local Government, 2015.

9. Bibby P, Brindley P. The 2011 Rural-Urban Classification For Small Area Geographies: A User Guide and Frequently Asked Questions (v1.0). Government Statistical Service, 2013.

10. Bibby P, Brindley P. Urban and Rural Area Definitions for Policy Purposes in England and Wales: Methodology (v1.0). Government Statistical Service, 2013.
