# Supplementary material for: Association of environmental and socioeconomic indicators with serious mental illness diagnoses identified from general practitioner practice data in England: A spatial Bayesian modelling study
Source: PLoS Med. 2022 Jun 30;19(6):e1004043. doi: 10.1371/journal.pmed.1004043 (PMC9286217; doi:10.1371/journal.pmed.1004043)
Supplement: S5 Table — All versions here include covariates a but they have different specifications to the random effects. Version 1 is the full model presented in the main paper. BYM, Besag-York-Mollié spatial prior model; CCG, Clinical Commissioning Group; DIC, Deviance Information Criterion; District, Local Authority District; MSOA, middle layer super output area; WAIC, Watanabe–Akaike information criterion. (DOCX) [file pmed.1004043.s005.docx]

Supplementary Material

S5 Table - Comparison of different versions of the full model. All versions here include covariates ^a^ but they have different specifications to the random effects. Version 1 is the full model presented in the main paper. (DIC - Deviance Information Criterion; WAIC - Watanabe–Akaike information criterion; MSOA – middle layer super output areas; District – Local Authority District; CCG – Clinical Commissioning Group; BYM - Besag-York-Mollié (BYM) spatial prior model)

| Version | Spatial scales (Random effects) | | | DIC | WAIC |
| --- | --- | --- | --- | --- | --- |
|  | MSOA | District | CCG |  |  |
| 1 | BYM | BYM | BYM | -68829 | -68562 |
| 2 | Exchangeable | Exchangeable | Exchangeable | -68454 | -68083 |
| 3 | Exchangeable | BYM | Exchangeable | -68437 | -68064 |
| 4 | BYM | BYM | Exchangeable | -68827 | -68551 |

^a^ The covariates are: Woodland, public green space, distance to nearest public green space, distance to the nearest public green space with a lake, distance to the nearest public green space with a river, distance to noise ≥75dB, distance to flood zone 3, annual mean of particulate matter 2.5, minority ethnic groups (Asian, Black, mixed) (%), 18-24 years old (%), 25-44 years old (%), 45-64 years old (%), ≥65 years old (%), Index of Multiple Deprivation 2015 (scores) of Crime domain, Income deprivation domain, Barriers to housing and services domain, Employment deprivation, Indoors subdomain, Adult skills subdomain, Urban and rural areas categories, English region categories
