# Supplementary material for: Association of environmental and socioeconomic indicators with serious mental illness diagnoses identified from general practitioner practice data in England: A spatial Bayesian modelling study
Source: PLoS Med. 2022 Jun 30;19(6):e1004043. doi: 10.1371/journal.pmed.1004043 (PMC9286217; doi:10.1371/journal.pmed.1004043)
Supplement: S6 Table — For explanation of the variables, see S4 Table. LSOA, Lower Layer Super Output Area; SD, standard deviation. (DOCX) [file pmed.1004043.s006.docx]

Supplementary Material

**Characterisation of all the covariates included in the models (average (SD)) in England and major conurbations by Lower Super Output Area (LSOA).**

The average area of public green space and woodland in major conurbations was lower than the national average (S6 Table). Public green spaces were on average below a 300 metres radius from the LSOA population weighted centroid at all spatial scales (S6 Table), whilst distance to green spaces with lakes varied between 29 km, at national level, and 7 km in Birmingham. Public green spaces with a river within their boundaries were more common, with one within a 2-km reach at national and major conurbations. The LSOAs of these areas were also closer to road traffic noise ≥75 dB, from 630 m (Greater London) to 1 km (Birmingham) than at national level (1.4 km) (S6 Table). Distance to flood zones 3 varied slightly between 700 m (national scale) to 930 m, in Greater London (S6 Table). The capital (13 μg m-3) and Birmingham (12 μg m-3) presented an annual concentration of PM_2.5_ higher than the national average (11 μg m-3) (S6 Table).

In terms of socio-demographic characteristics, more than a third of the population living in Greater London belonged to ethnic minorities (36%), with all major conurbations presenting a higher percentage of people belonging to this group compared with national average (14%), with exception of Newcastle, with the lowest value (6%) (S6 Table). In terms of age class, most of the major conurbations mirrored the national results, with the exception of Greater London. People aged between 25-44 years old were at higher percentage, and people older than 65 years old were in a lower percentage in Greater London than in the rest of England and other major conurbations (S6 Table). Compared to the national values for the different domains and subdomains of the English Indices for Deprivation 2015, Newcastle presented lower scores (less deprived) in crime, barriers to housing and services domains and indoors subdomain (S6 Table). Greater London had lower scores of employment deprivation domain and adult skills subdomain (S6 Table).”

S6 Table - Characterisation of all the covariates included in the models (average (SD)) in England and major conurbations by Lower Super Output Area (LSOA). For explanation of the variables see Table S4.

|  | National | Major Conurbations | | | | |
| --- | --- | --- | --- | --- | --- | --- |
|  | England | Greater London | Birmingham | Liverpool and Manchester | Leeds | Newcastle |
|  | Mean (SD) | | | | | |
| Woodland area (ha) | 38 (249) | 3.52 (14.55) | 3.64 (18.61) | 4.68 (12.66) | 6.58 (20.21) | 4.71 (16.78) |
| Public green space (ha) | 9.37 (28.31) | 5.35 (17.23) | 6.06 (22.78) | 6.46 (13.86) | 6.47 (15.53) | 6.91 (14.01) |
| Distance to nearest public green space (km) | 0.24 (0.21) | 0.20 (0.12) | 0.24 (0.13) | 0.21 (0.12) | 0.20 (0.12) | 0.20 (0.12) |
| Distance to the nearest public green space with a lake (km) | 29.14 (21.75) | 20.10 (7.28) | 7.30 (4.59) | 12.03 (8.45) | 16.98 (7.27) | 26.56 (5.14) |
| Distance to the nearest public green space with a river (km) | 2.18 (2.46) | 1.88 (1.67) | 1.94 (1.90) | 1.74 (1.84) | 2.01 (1.93) | 1.77 (1.94) |
| Distance to noise ≥75dB (km) | 1.40 (2.06) | 0.63 (0.49) | 1.01 (0.66) | 0.84 (0.62) | 0.91 (0.62) | 0.80 (0.53) |
| Distance to flood zone 3 (km) | 0.70 (0.71) | 0.93 (0.95) | 0.85 (0.57) | 0.72 (0.57) | 0.73 (0.49) | 0.90 (0.56) |
| Annual mean of particulate matter 2.5 (PM_2.5_) (μg m^-3^) | 11.06 (1.61) | 13.28 (1.35) | 12.01 (0.88) | 10.26 (0.75) | 10.46 (0.81) | 9.71 (0.40) |
| Minority ethnic groups (Asian, Black, mixed) (%) | 13.78 (4.05) | 35.69 (9.89) | 27.20 (10.26) | 15.09 (6.15) | 20.62 (10.34) | 5.95 (3.28) |
| 18-24 years old (%) | 9.08 (6.17) | 9.63 (4.06) | 9.84 (6.18) | 10.13 (7.60) | 10.63 (9.70) | 10.53 (9.05) |
| 25-44 years old (%) | 27.29 (7.61) | 34.31 (9.07) | 27.25 (5.3) | 27.6 (6.75) | 28.43 (6.02) | 25.05 (5.24) |
| 45-64 years old (%) | 25.68 (5.61) | 22.01 (0.86) | 23.58 (5.40) | 25.28 (5.64) | 23.82 (6.36) | 26.79 (5.57) |
| ≥65 years old (%) | 16.64 (7.24) | 11.90 (5.28) | 15.81 (6.33) | 15.43 (6.03) | 14.36 (5.97) | 16.75 (6.12) |
| Crime domain scores | 0.00 (0.78) | 0.46 (0.62) | 0.22 (0.64) | 0.23 (0.72) | 0.40 (0.69) | -0.29 (0.72) |
| Income deprivation domain scores | 0.15 (0.10) | 0.16 (0.09) | 0.21 (0.12) | 0.20 (0.13) | 0.18 (0.12) | 0.19 (0.12) |
| Barriers to housing and services domain scores | 21.69 (10.57) | 28.34 (9.85) | 25.43 (11.53) | 15.56 (7.88) | 18.52 (6.88) | 17.42 (7.31) |
| Employment deprivation scores | 0.12 (0.08) | 0.11 (0.06) | 0.16 (0.08) | 0.17 (0.10) | 0.15 (0.08) | 0.17 (0.09) |
| Indoors subdomain scores | 0.00 (0.86) | 0.06 (0.60) | 0.38 (0.66) | 0.17 (0.71) | 0.55 (0.71) | -1.01 (0.80) |
| Adult skills subdomain scores | 0.31 (0.11) | 0.27 (0.11) | 0.39 (0.12) | 0.35 (0.13) | 0.36 (0.13) | 0.35 (0.12) |
