# Supplementary material for: Association of environmental and socioeconomic indicators with serious mental illness diagnoses identified from general practitioner practice data in England: A spatial Bayesian modelling study
Source: PLoS Med. 2022 Jun 30;19(6):e1004043. doi: 10.1371/journal.pmed.1004043 (PMC9286217; doi:10.1371/journal.pmed.1004043)
Supplement: S1 Fig — CCG, Clinical Commissioning Group; District, Local Authority District; MSOA, middle layer super output area; SD, standard deviation. (DOCX) [file pmed.1004043.s007.docx]

Supplementary Material

S1 Figure **-** posterior distributions of the random effect standard deviations (Std. Dev.). (MSOA – middle layer super output areas; District – Local Authority District; CCG – Clinical Commissioning Group)

**
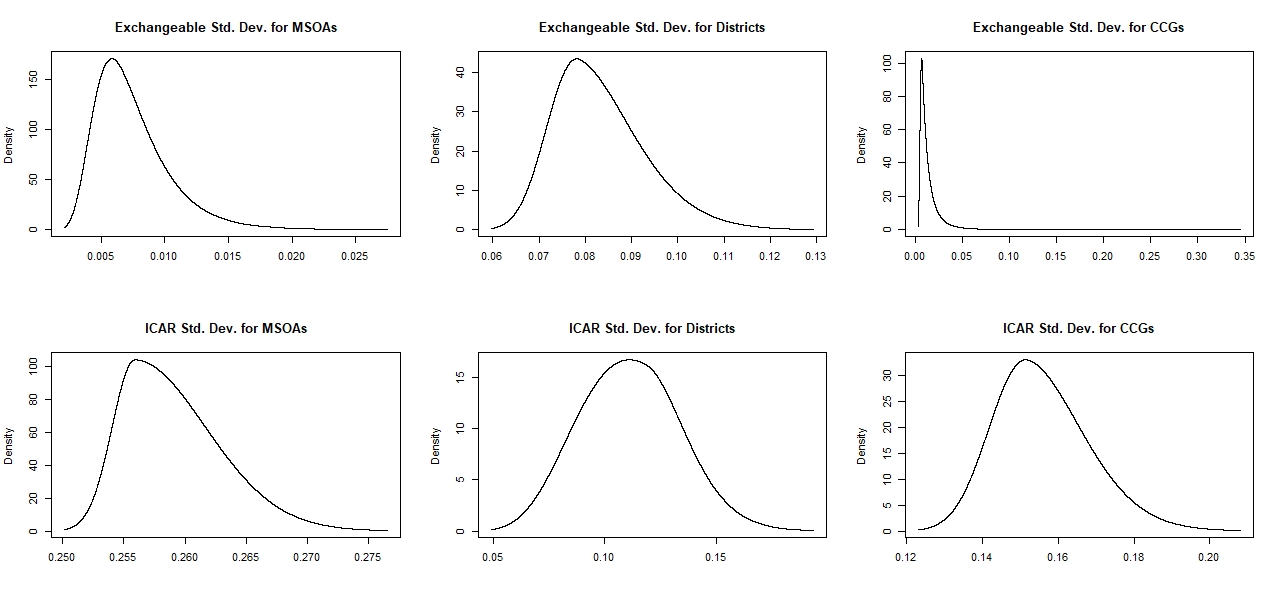
**
